# Persistent Type I Interferon Signaling Impairs Innate Lymphoid Cells During HIV-1 Infection Under Suppressive ART

**DOI:** 10.3390/v17081099

**Published:** 2025-08-08

**Authors:** Runpeng Han, Haisheng Yu, Guangming Li, Lishan Su, Liang Cheng

**Affiliations:** 1 State Key Laboratory of Virology and Biosafety, Department of Infectious Diseases, Medical Research Institute, Frontier Science Center for Immunology and Metabolism, Zhongnan Hospital of Wuhan University, Wuhan University, Wuhan 430071, China; harrishan@hotmail.com; 2 Lineberger Comprehensive Cancer Center, Department of Microbiology and Immunology, University of North Carolina at Chapel Hill, Chapel Hill, NC 27599, USA; yuhaisheng@gzhmu.edu.cn (H.Y.); guangming.li@ihv.umaryland.edu (G.L.); 3 Infectious Disease Center, Guangzhou Eighth People’s Hospital, Guangzhou Medical University, Guangzhou 510440, China; 4 Division of Virology, Pathogenesis, and Cancer, Institute of Human Virology, Department of Pharmacology, University of Maryland School of Medicine, Baltimore, MD 21201, USA; 5 Center for AIDS Research, Zhongnan Hospital of Wuhan University, Wuhan 430071, China

**Keywords:** human immunodeficiency virus, type-I interferon, immunopathogenesis, innate lymphoid cells, humanized mice

## Abstract

Persistent type I interferon (IFN-I) signaling compromises adaptive anti-HIV-1 T cell immunity and promotes viral reservoir persistence, yet its effects on innate lymphoid cells during chronic infection remain unclear. Through integrated single-cell RNA sequencing and functional validation in HIV-1-infected humanized mice with combination antiretroviral therapy (cART) and IFN-I signaling blockade, we reveal IFN-I-induced dysfunction of natural killer (NK) cells and group 3 innate lymphoid cells (ILC3s). Mechanistically, the IFN-I-CD9 axis drives NK cells toward a decidual NK cell-like phenotype, impairing their cytotoxic activity. Furthermore, IFNAR blockade rescues ILC3 functionality, which is critical for IL-17/IL-22-mediated antimicrobial defense and mucosal barrier maintenance. Our study delineates IFN-I-driven immunosuppression across innate lymphocyte compartments and proposes the targeted modulation of this pathway to enhance antiviral and mucosal immunity in HIV-1 management.

## 1. Introduction

Significant advancements in the management of HIV-1 infection have been achieved through combined antiretroviral therapy (cART) [1,2,3]. However, despite its ability to effectively suppress viral replication, cART fails to fully resolve the immune dysfunction and chronic inflammation associated with HIV-1 infection [4]. This persistent immune dysregulation underscores the need for a deeper understanding of the underlying mechanisms driving both innate and adaptive immune dysfunction during HIV-1 infection.

Innate lymphocytes demonstrate functional parallels with adaptive T lymphocytes, where natural killer (NK) cells mirror CD8^+^ cytotoxic T cell activity and innate lymphoid cells (ILCs) recapitulate CD4^+^ T helper cell functions [5]. During HIV-1 infection, NK cells orchestrate antiviral responses through dual mechanisms: (1) cytokine secretion (e.g., IFN-γ and TNF-α) that establishes antiviral states, and (2) direct cytolytic activity mediated by perforin- and granzyme-containing cytotoxic granules that eliminate infected cells and restrain viral dissemination [6]. Notably, multiple cohort studies have established correlations between NK cell functional competence and two key clinical outcomes: resistance to HIV acquisition [7] and spontaneous viral control in untreated individuals [8,9,10]. However, chronic HIV-1 infection, even in virologically suppressed patients recei9ving combination antiretroviral therapy (cART), induces NK cell dysfunction characterized by three hallmark features: (1) upregulated inhibitory receptors (e.g., KLRG1 [11] and KIR [12]), (2) downregulated activating receptors (e.g., CD16 [13] and NKG2D [14]), and (3) impaired responsiveness to activation signals [11]. These pathophysiological alterations underscore the necessity of elucidating the mechanisms underlying HIV-1-induced NK cell impairment, a critical prerequisite for developing targeted immunotherapies to restore antiviral function and improve long-term clinical outcomes in cART-treated patients.

The classification of ILCs into three subsets (ILC1s, ILC2s, and ILC3s) mirrors the functional specialization of adaptive T helper (Th) cell lineages, with ILC1s producing interferon-γ (IFN-γ) to combat intracellular pathogens, ILC2s secreting type 2 cytokines (IL-4, IL-5, IL-9, and IL-13) for anti-helminth immunity and tissue repair, and ILC3s generating IL-17 and IL-22 to maintain gut mucosal barrier integrity through antimicrobial defense and epithelial regeneration [5]. In HIV infection, the loss of ILCs during the acute viremic phase has been observed, and this can persist into the chronic stage of the disease, even in individuals receiving cART [15]. The rapid depletion of IL-22-producing ILC3s during acute viremia disrupts intestinal epithelial tight junctions, promotes microbial translocation, and drives systemic inflammation [16]. This ILC3–gut axis dysfunction highlights its central role in HIV pathogenesis, suggesting that therapeutic strategies targeting ILC3 reconstitution could potentially ameliorate barrier defects and chronic inflammation in people living with HIV (PLWH).

Previous investigations from our group and others have established that the therapeutic blockade of IFN-I signaling through anti-interferon-α/β receptor (IFNAR) blocking antibodies reverses CD4^+^ T cell depletion, enhances CD8^+^ T cell antiviral activity, and prolongs viral suppression following cART interruption [17,18,19,20]. However, the immunological consequences of IFNAR inhibition on innate lymphocyte populations, particularly NK cells and ILCs, remain undefined. Through systematic single-cell transcriptomic analysis combined with functional validation in HIV-1-infected humanized mice under cART suppression, we demonstrate that IFNAR blockade restores the cytotoxic potential of NK cells and rescues the cytokine-producing capacity of ILC3s. These findings reveal the previously unrecognized IFN-I-mediated mechanisms driving innate immune dysfunction in chronic HIV-1 infection.

## 2. Results

### 2.1. Similarity in Innate Immune Cell Subtypes Between Humanized Mouse Spleens and Human Spleen-Derived Counterparts

**Figure 1:**
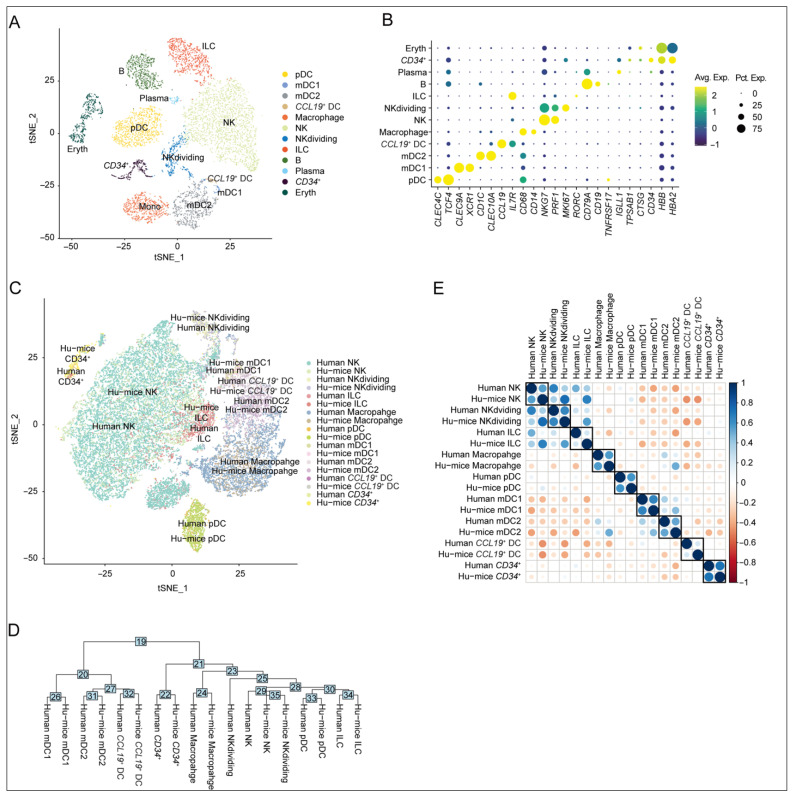
Similarity in human innate immune cell subtypes from humanized mouse spleens and human spleens. (**A**) t-SNE plot of splenic hCD45^+^CD3^-^CD19^-^ immune cells from mock-treated mice, annotated by cell type. (**B**) Marker gene expression for each cell population (dot size: expression frequency; color: scaled average expression). (**C**) Integrated t-SNE of innate immune subsets from spleens of both species, colored by cell type and source. (**D**) Hierarchical clustering dendrogram based on PCA distances. Distance values in the PCA space were annotated on the nodes. (**E**) Pearson correlation matrix of transcriptomes between humanized mice and human immune subsets. The value of Pearson’s correlation coefficient was shown on a color scale, and the absolute value was shown on a size scale.

Our previous single-cell RNA-seq results demonstrated the successful reconstitution of major human innate immune subsets in humanized NRG mice (hu-mice) 12 weeks after the transplantation of human CD34^+^ hematopoietic stem cells [21]. To assess the translational relevance of this model, we performed comparative transcriptomic profiling between innate immune cells isolated from hu-mice spleens (Figure 1A,B) and their counterparts derived from cold-stored human spleens [22]. Through t-SNE-based dimensionality reduction and phylogenetic tree reconstruction of scRNA-seq datasets, we observed that innate immune subsets in hu-mice exhibited lineage-dependent clustering patterns with substantial overlap between murine-reconstituted populations and their human splenic counterparts (Figure 1C,D). Subsequent Pearson correlation analysis further confirmed strong transcriptional concordance in key innate immune populations, including natural killer (NK) cells and innate lymphoid cells (ILCs), between hu-mice and human reference samples (Figure 1E). These analyses collectively establish the humanized mouse model as a physiologically representative system for investigating human innate immunity.

### 2.2. Single-Cell Transcriptomic Landscape Reveals HIV-1- and IFN-I-Driven Transcriptional Reprogramming of Innate Immune Cells

**Figure 2:**
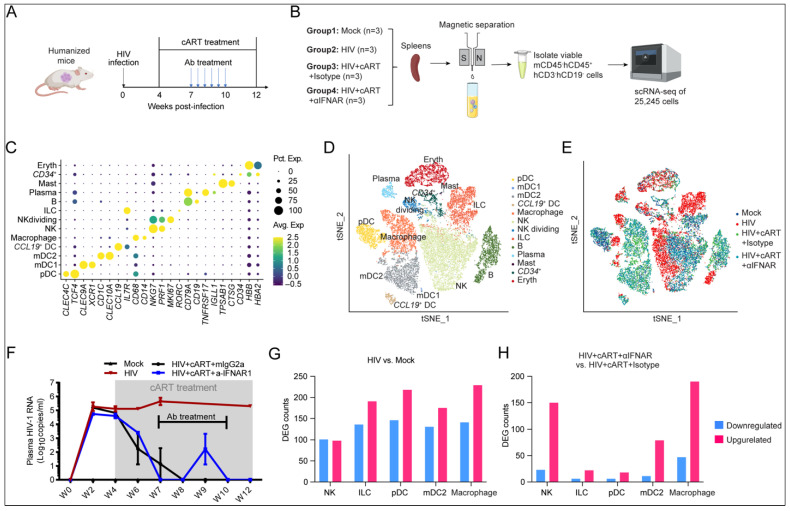
Single-cell profiling of human splenic innate immune cells in humanized mice with HIV-1 infection under cART and IFNAR blockade. (**A**) Experimental timeline: humanized mice infected with HIV-1 received cART from 4 to 12 weeks post-infection (wpi). Mice were administered α-IFNAR1 or isotype control (mIgG2a) twice weekly by i.p. injection from 7 to 10 wpi and sacrificed at 12 wpi. (**B**) Workflow for enriching human innate immune cells (hCD45^+^CD3^−^CD19^−^) from splenocytes and subsequent scRNA-seq analysis. (**C**) Dot plot depicting cluster-defining marker gene expression. Dot size indicates the percentage of cells expressing the gene; color intensity reflects scaled average expression. (**D**,**E**) t-SNE visualization of 25,245 hCD45^+^CD3^−^CD19^−^ cells, colored by annotated cell types (**D**) or experimental groups (**E**). (**F**) HIV-1 RNA levels in the plasma of humanized mice treated in (**A**). (**G**,**H**) Differentially expressed genes (DEGs) in specified comparisons. Upregulated: avg. log2FC > 0.25, adj. *p* < 0.05; downregulated: avg. log2FC < −0.25, adj. *p* < 0.05.

Next, we employed the humanized mouse model to investigate HIV-1-mediated perturbations in innate lymphoid cell function and their modulation by IFN-I signaling in vivo. To achieve this, we performed scRNA-seq on human hCD45^+^CD3^−^CD19^−^ splenocytes across four experimental groups (n = 3/group): (1) mock-infected controls, (2) HIV-1-infected mice, (3) HIV-1-infected mice treated with cART and isotype antibody, and (4) HIV-1-infected mice treated with cART and anti-IFNAR antibody (Figure 2A,B). After stringent quality control, 25,245 high-confidence single-cell transcriptomes were retained for analysis. Unsupervised clustering identified 13 distinct populations encompassing NK cells, ILCs, macrophages, dendritic cell subsets (pDCs, mDC1, mDC2, and *CCL19*^+^ DC), mast cells, and minor contaminants (CD34^+^ progenitors, B-lineage cells, and erythroid cells) (Figure 2C–E; Supplemental Table S1).

As previously reported [17], HIV-1 infection in humanized mice leads to persistent viremia, which can be suppressed by cART (Figure 2F). Differential gene expression (DEG) analysis demonstrated that HIV-1 infection induced significant transcriptional reprogramming across all innate immune compartments (Figure 2G, Supplemental Table S2), consistent with our previous report [21]. Gene Ontology enrichment analysis of upregulated genes revealed the profound activation of type I interferon signaling pathways, antiviral defense mechanisms, and negative regulation of viral replication across diverse cell types (Supplemental Table S3). Previous reports have shown that despite the efficient suppression of HIV-1 replication with cART, abnormally elevated IFN-I signaling persists in certain patients even under extensive cART [23,24], as well as in HIV-1-infected humanized mouse models [17]. Our scRNA-seq data further demonstrated that while cART successfully suppressed HIV-1 replication and reduced infection-induced transcriptional changes (Supplemental Tables S2 and S3), the pharmacological blockade of IFNAR signaling during cART administration additionally reversed HIV-associated transcriptional profiles in innate immune populations, particularly NK cells and innate lymphoid cells (ILCs) (Figure 2H, Supplemental Tables S2 and S3). These collective findings indicate that persistent IFN-I signaling during chronic infection continues to regulate innate immune functionality even following successful viral suppression.

### 2.3. IFNAR Blockade Reverses Transcriptional Dysregulation of NK Cells in Chronic HIV-1 Infection Under cART

**Figure 3:**
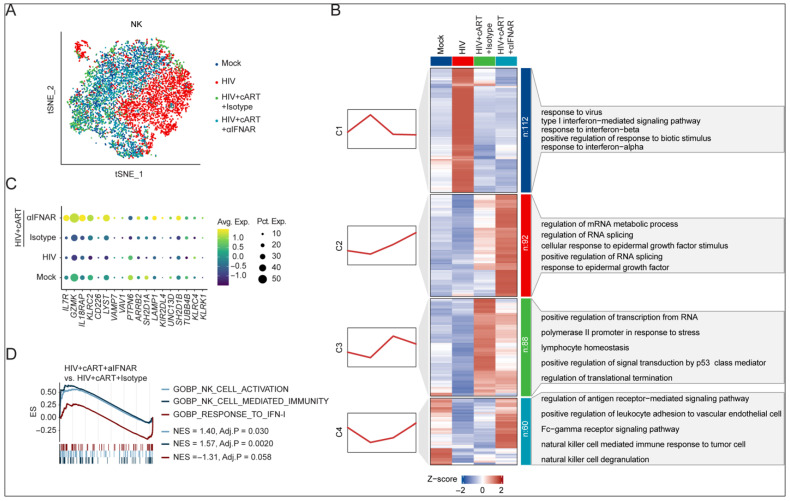
IFNAR blockade reverses HIV-1-induced NK cell transcriptional dysregulation refractory to cART. (**A**) t-SNE projection of splenic NK cells, colored by experimental groups. (**B**) Mfuzz-clustered heatmap of NK cell transcriptional dynamics. Red trendlines denote expression patterns; enriched pathways are annotated. (**C**) Expression of activation/effector genes across groups (dot size: expression frequency; color: scaled average expression). (**D**) GSEA of IFNAR-blockaded vs. isotype-treated NK cells from HIV-1-infected mice with cART.

To investigate the mechanisms by which persistent IFN-I signaling drives NK cell dysfunction during HIV-1 infection under cART, we investigated the transcriptomic changes in splenic NK cells from hu-mice across four experiment conditions (Figure 3A). Through integrated differential expression and unsupervised clustering analysis, we identified four distinct transcriptional modules (C1–C4) that delineated HIV-1- and treatment-dependent molecular signatures. Module C1 contained interferon-stimulated genes (ISGs) that were upregulated during chronic HIV-1 infection but subsequently suppressed by cART and IFNAR blockade (Figure 3B and Supplemental Table S4). In hu-mice treated with cART alone or cART plus IFNAR blockade, we observed increased activity of RNA processing-related genes (C2 and C3, Figure 3B). This aligns with prior findings showing that HIV-positive individuals with successful viral suppression on cART exhibit higher RNA processing gene activity compared to uninfected individuals or those without viral suppression [25]. Notably, module C4 revealed critical functional deficits in HIV-infected mice, showing the suppression of NK cell effector pathways, including NK cell degranulation and Fc-gamma receptor signaling pathways, that were specifically restored only by cART combined with anti-IFNAR treatment (Figure 3B). This transcriptional change aligns with the clinical observations of persistent NK cell dysfunction in virologically suppressed PLWH [11,12,13,14] and suggests therapeutic potential for IFN-I modulation. At the individual gene level, we observed the significant downregulation of NK cell survival and effector markers (e.g., *IL7R*, *GZMK*, *IL18RAP*, and *KLRC2*) in HIV-infected mice that persisted through cART monotherapy (Figure 3C). Strikingly, combining therapy with cART and IFNAR-blocking antibody restored the expression of these critical genes (Figure 3C). Complementary gene set enrichment analysis (GSEA) confirmed that IFNAR blockade simultaneously attenuated the IFN-I signaling pathways while enhancing the gene signatures associated with NK cell activation and NK cell-mediated immunity (Figure 3D).

### 2.4. HIV-Induced CD9^+^ dNK-like Cells Persist via IFN-Dependent Mechanisms Under cART

**Figure 4:**
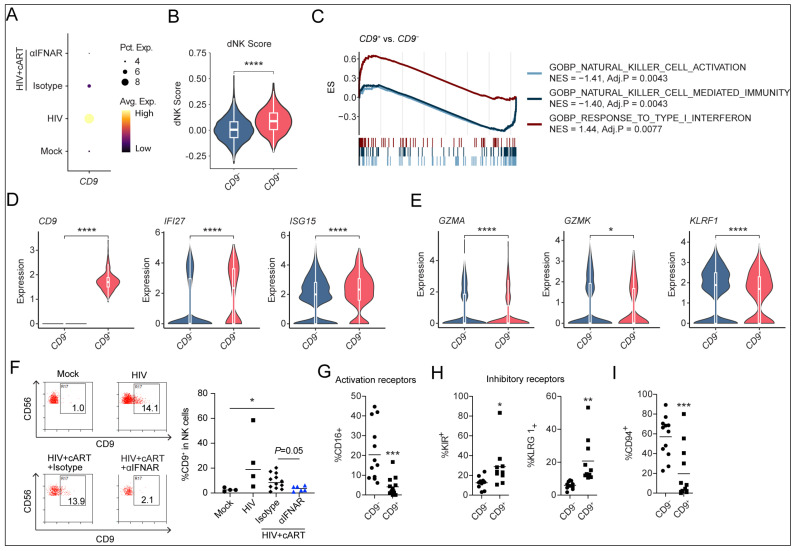
HIV-induced CD9^+^ dNK-like cells persist via IFN-dependent mechanisms under cART. (**A**) CD9 expression in NK cells across groups (dot size: expression frequency; color: scaled average expression). (**B**) Violin–box plots comparing dNK signature scores in CD9^+^ vs. CD9^−^ NK cells from all groups of mice. (**C**) GSEA of CD9^+^ vs. CD9^−^ NK cells from all groups of mice. (**D**) Violin–box plots comparing *CD9*, *IFI27*, and *ISG15* expression in *CD9*^+^ vs. *CD9*^−^ NK cells from all groups of mice. (**E**) Violin–box plots comparing expression of cytotoxic effectors (*GZMA*, *GZMK*, and *KLRF1*) in *CD9*^+^ vs. *CD9*^−^ NK cells from all groups of mice. (**F**) Humanized mice were treated as in Figure 2A. Mice were sacrificed at 12 wpi. Representative FACS plots (**left**) and summarized data (**right**) show the percentage of CD9^+^ NK cells across 4 groups. (**G**–**I**) Expression of activation (**G**) and inhibitory (**H**) receptors, and CD94 (**I**) in CD9^+^ vs. CD9^−^ NK cells. Statistical analyses by *t*-test (**D**,**E**,**G**–**I**) or Brown–Forsythe and Welch ANOVA tests, followed by Dunnett’s T3 multiple comparisons test (**F**). * *p* < 0.05, ** *p* < 0.01, *** *p* < 0.001, **** *p* < 0.0001.

Previous studies have reported that CD9^+^ decidual NK (dNK) cells exhibit reduced cytotoxicity [26,27], and the inhibition of CD9 has been shown to suppress HIV replication [28]. Our scRNA-seq data revealed that HIV-1 infection increased *CD9* expression in NK cells, and cART failed to restore *CD9* expression to the baseline levels observed in mock-infected mice (Figure 4A). Notably, this shift was reversed by IFNAR blockade (Figure 4A). The gene expression profiling of *CD9*^+^ NK cells demonstrated a significant increase in dNK-like properties compared to their *CD9*^−^ counterparts (Figure 4B). Intriguingly, GSEA revealed that *CD9*^+^ NK cells exhibited an elevated IFN-I response but a decrease in the pathways associated with NK cell activation and NK cell-mediated immunity (Figure 4C). Consistent with this, *CD9*^+^ NKs showed a statistically significant yet modest upregulation of IFN-I stimulated genes (e.g., *IFI27* and *ISG15*) and downregulation of NK effector genes (e.g., *GZMA*, *GZMK*, and *KLRF1*) (Figure 4D,E).

To experimentally validate these findings, we assessed whether CD9 surface expression on NK cells was induced by HIV-1 infection and IFN-I signaling by flow cytometry (Figure S1). We found that the median percentage of CD9^+^ NK cells increased by approximately 9.8-fold in the HIV-1-infected group compared with the mock group (Figure 4F). While cART slightly reduced the percentage of CD9^+^ NK cells, the combination of cART and IFNAR blockade completely restored the CD9^+^ NK cell levels to baseline (Figure 4F). Given that NK cell function is regulated by a balance of activating and inhibitory receptors [29], we further analyzed receptor expression using flow cytometry. We observed that CD9^+^ NK cells exhibited higher surface levels of activating receptor CD16 compared to CD9^−^ NK cells (Figure 4G). Conversely, inhibitory receptors such as KIR and KLRG1 were upregulated in CD9^+^ NK cells (Figure 4H), suggesting a less activated phenotype. Moreover, CD94, a co-receptor for the NKG2 family, which can form both activating and inhibitory receptors for HLA-E [30,31], was also downregulated in CD9^+^ NK cells (Figure 4I), implicating their blunted signal transduction upon HLA-E engagement.

In summary, by integrating bioinformatic and flow cytometry approaches, we identified an IFN-CD9 axis in NK cells during HIV-1 infection that likely contributes to their impaired activation and reduced cytolytic antiviral activity.

### 2.5. IFN-I Signaling Drives HIV-1-Induced Dysfunction of ILC3s

**Figure 5:**
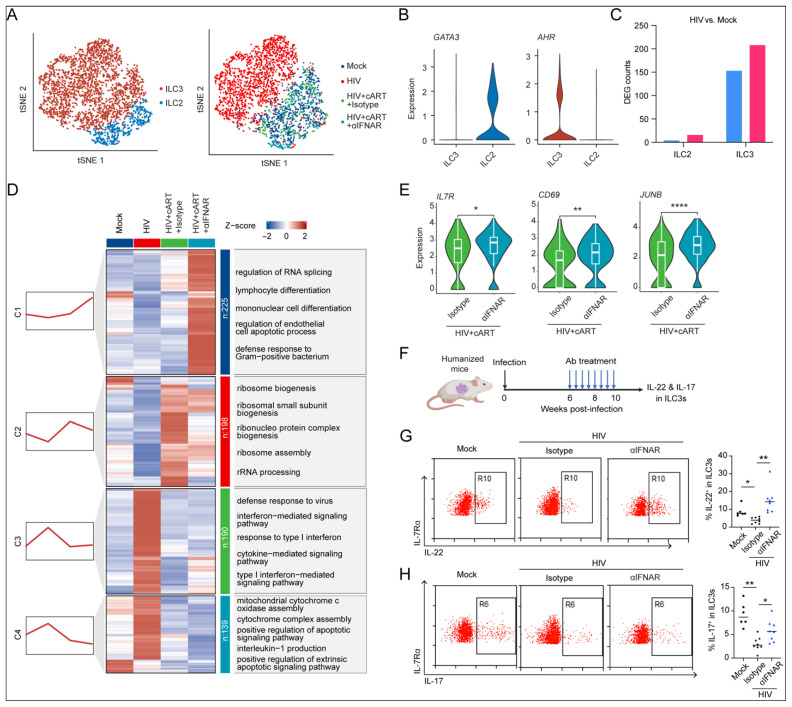
**IFNAR blockade restores ILC3 homeostasis and cytokine production.** (**A**) t-SNE plot of ILCs, colored by subtypes (**left**) or groups (**right**). (**B**) Violin plot showing the expression of *GATA3* and *AHR* in ILC2s and ILC3s. (**C**) Number of DEGs in specified comparisons. Upregulated (pink): avg. log2FC > 0.25, adj. *p* < 0.05; downregulated (blue): avg. log2FC < −0.25, adj. *p* < 0.05. (**D**) Mfuzz-clustered heatmap of NK cell transcriptional dynamics. Red trendlines denote expression patterns; enriched pathways are annotated. (**E**) Violin–box plots comparing *IL7R*, *CD69*, and *JUNB* expression across ILC3s from 4 groups. (**F**) Schematic diagram of experiment design. Hu-mice infected with HIV-1 were treated with α-IFNAR1 antibody or isotype control twice a week through week 6 to week 10. Mice were sacrificed at 10 wpi. The splenic cells were collected and stimulated with PMA and ionomycin ex vivo, and the expression of IL-22 and IL-17 in human ILC3s was detected by flow cytometry. (**G**,**H**) Representative FACS plots and summarized data show the frequencies of IL-22^+^ (**G**) and IL-17^+^ (**H**) ILC3s across 4 groups. Statistical analyses by Brown–Forsythe and Welch ANOVA tests, followed by Dunnett’s T3 multiple comparisons test (**G**,**H**). * *p* < 0.05, ** *p* < 0.01, ****p < 0.0001.

ILCs serve as crucial regulators of mucosal immunity, orchestrating both antimicrobial defense and tissue repair through cytokine-mediated mechanisms [5]. Building upon previous reports of HIV-1-associated ILC depletion [15,32,33], we investigated the IFN-I-mediated dysregulation of ILC subsets during chronic infection. Our scRNA-seq identified two ILC populations in the spleens of humanized mice: *GATA3*^+^ ILC2s and *AHR*^+^ ILC3s (Figure 5A,B). The DEG analysis revealed predominant HIV-1-induced perturbations in the ILC3 compartment (Figure 5C), prompting our focused investigation on this subset.

ILC3s are characterized by their production of IL-17 and IL-22, cytokines critical for anti-bacterial immunity [34] and maintaining intestinal homeostasis [35]. We first surrogated the gene expression patterns in ILC3s under different infection and therapy contexts (Figure 5D and Supplemental Table S5). Notably, we observed that only cART combined with IFNAR blockade enhanced antimicrobial and tissue-regulatory transcriptional programs (module C1) (Figure 5D). This finding mirrors clinical observations of persistent ILC dysfunction in virologically suppressed individuals [15] and suggests therapeutic potential for IFN-I modulation. Moreover, IFNAR blockade synergized with cART to downregulate proapoptotic pathways in ILC3s (module C4) (Figure 5D), consistent with our prior findings that pDC depletion rescues ILC survival [32]. In addition to survival-related genes, IFNAR blockade also significantly downregulates IL-1β production- and inflammasome-activation-related genes (module C4) (Figure 5D and Supplemental Table S5), which is reported to be a major cause of CD4^+^ T cell loss during HIV-1 infection [36], suggesting that IFNAR blockade may rescue ILC3s via inhibiting pyroptosis in addition to apoptosis. Moreover, similar to what is observed in NK cells, hu-mice that received cART alone and cART+IFNAR blockade showed the activation of genes related to RNA processing and RNA translation in splenic ILC3s (module C2) (Figure 5D). The therapeutic efficacy was further evidenced by the upregulated expression of (1) IL-7 receptor (*IL7R*), critical for lymphocyte homeostasis, (2) activation marker *CD69*, and (3) *JUNB* transcription factor, whose deficiency drives ILC3 dysfunction and intestinal inflammation [37] (Figure 5E).

To functionally validate these observations, humanized mice were infected with HIV-1 followed by the administration of either IFNAR-blocking antibody or isotype control from weeks 6 to 10 post-infection (Figure 5F). Following euthanasia at the experimental endpoint (week 10), we quantitatively assessed the cytokine production capacity of splenic ILC3s through PMA/ionomycin stimulation coupled with multiparametric flow cytometry (Figure S2). Comparative analysis revealed the significant suppression of IL-22^+^ and IL-17^+^ ILC3 frequencies in HIV-1-infected mice versus the uninfected controls (Figure 5G,H). Importantly, IFNAR blockade restored cytokine production, demonstrating the reversal of HIV-induced functional impairment (Figure 5G,H).

Collectively, these findings establish IFN-I signaling as a key driver of ILC3 functional impairment during chronic HIV-1 infection, with targeted IFNAR inhibition showing the potential to reverse this immunopathological process.

## 3. Discussion

Accumulating evidence, including our prior work, has established that sustained IFN-I signaling during chronic HIV-1 infection exacerbates CD4^+^ T cell depletion, impairs adaptive immunity, and promotes viral persistence [17,18]. While these studies focused on adaptive immune compartments, the immunological consequences of IFN-I blockade on innate lymphoid populations remained unexplored. Our current investigation using humanized mouse models demonstrates that IFNAR inhibition restores both NK cell and ILC3 functionality, revealing previously unrecognized IFN-I-mediated mechanisms of innate immune dysregulation. These findings provide critical mechanistic insights into how targeted immunomodulation could complement existing therapies to address persistent immune dysfunction—a major obstacle to achieving HIV remission.

NK cells play dual roles in HIV-1 immunity through direct cytolytic activity and immunoregulatory cytokine production [38]. Here, we demonstrated that cART in HIV-1-infected humanized mice fails to restore NK cell function, a finding consistent with observations from virologically suppressed PLWH [14]. The restoration of canonical NK functionality through IFNAR blockade raises intriguing questions about IFN-I’s role in promoting immunotolerant NK differentiation, a process evolutionarily conserved in maternal–fetal tolerance [39]. Moreover, given the established links between persistent NK dysfunction and increased cancer incidence [40] or opportunistic infections [41] in PLWH, our findings warrant clinical investigation into whether IFN-I modulation could reduce these comorbidities.

Regarding ILC3s, while previous studies reported numerical restoration through early cART initiation [15] or pDC depletion [32], we demonstrate that IFNAR blockade combined with cART functionally rescues this population by restoring IL-17/IL-22 production. This functional recovery suggests potential benefits for mucosal barrier integrity, given the established correlation between ILC3 loss, gut barrier disruption, and microbial translocation in chronic HIV-1 infection [16]. However, current humanized mouse models’ limitations in recapitulating gut-associated lymphoid tissue (GALT) and human microbiota [42,43] preclude direct assessment of these physiological outcomes. Next-generation humanized models with improved GALT reconstruction could bridge this gap while enabling microbiome analysis. Furthermore, our study’s focus on ILC3s necessitates future investigations into other ILC subsets (ILC1s/ILC2s), given their distinct roles in antiviral defense and tissue homeostasis [44].

Collectively, our findings delineate a central pathogenic role of chronic IFN-I signaling in driving innate lymphoid cell dysfunction during HIV-1 persistence. The dual restoration of NK cell cytotoxicity and ILC3 effector functions through IFNAR blockade not only advances our understanding of HIV-1 immunopathogenesis but also establishes a therapeutic paradigm for addressing the multifaceted immune dysfunction in PLWH. Future studies should explore the translational potential of combining IFN-I modulation with existing regimens to restore antiviral immunity and improve clinical outcomes.

## 4. Materials and Methods

### 4.1. Generation of Hu-Mice

NRG mice (NOD Rag2^−/−^ γc^−/−^) were obtained from The Jackson Laboratory (ME, USA). Humanized NRG-hu HSC mice were generated through the intrahepatic injection of 2 × 10^5^ CD34^+^ hematopoietic progenitor cells isolated from human fetal liver tissue into neonatal (2–5 days postpartum) NRG pups, as previously described [21]. The engraftment efficiency was monitored by quantifying human CD45^+^ leukocytes in peripheral blood using flow cytometry at 10–12 weeks post-transplantation. Animals demonstrating over 20% human reconstitution (human CD45^+^ cells) were included in subsequent experiments. All mice were kept in a specific pathogen-free environment.

### 4.2. HIV-1 Infection of Humanized Mice

The HIV-1 JR-CSF infectious molecular clone (pYK-JRCSF) was acquired from the NIH AIDS Reagent Program (NIH, MD, USA, catalog #2708). Recombinant virus was generated by transfecting human embryonic kidney 293T cells (ATCC CRL-3216) with pYK-JRCSF plasmid DNA. Mice were anesthetized and infected with 10 ng of HIV-1 p24 equivalent (JR-CSF strain) via retro-orbital injection.

### 4.3. Combination Antiretroviral Therapy

We prepared medicated food pellets by incorporating three antiretroviral drugs—emtricitabine (FTC, an NRTI, Gilead Sciences, CA, USA), tenofovir disoproxil fumarate (TDF, an NRTI from Truvada, Gilead Sciences, CA, USA), and raltegravir (RAL, an integrase inhibitor from Isentress, Merck, NJ, USA)—into standard rodent chow following an established protocol we published previously [17]. First, we crushed commercial tablets of each drug into a fine powder, which was then thoroughly homogenized with TestDiet 5B1Q (a modified LabDiet 5058 containing amoxicillin) before being pressed into ½-inch irradiated pellets. The final drug concentrations in the feed were 4800 mg/kg for raltegravir (targeting ~768 mg/kg/day), 1560 mg/kg for tenofovir disoproxil (targeting ~250 mg/kg/day), and 1040 mg/kg for emtricitabine (targeting ~166 mg/kg/day). These elevated concentrations were selected to ensure adequate drug exposure and therapeutic efficacy in our experimental model while maintaining the physical stability and palatability of the formulated diet.

### 4.4. IFNAR Blocking Antibody Generation and Treatments

Anti-IFNAR1 blocking antibodies were generated as previously described [17]. Antibodies were administered intraperitoneally twice a week. The initial dose was 400 μg per mouse, followed by 200 μg per mouse for subsequent treatments.

### 4.5. Isolation of mCD45^–^hCD3^–^hCD19^–^hCD45^+^ Splenocytes from Humanized Mice

Splenocytes (2 × 10^6^ cells) were isolated from humanized mice three weeks after HIV-1 infection or mock treatment. For the initial negative selection, cells were incubated on ice for 20 min in staining buffer (1× PBS with 2% FBS and 2 mM EDTA) containing biotin-conjugated anti-mouse CD45 (0.5 μg/10^6^ cells), anti-human CD3 (0.5 μg/10^6^ cells), and anti-human CD19 (0.5 μg/10^6^ cells). Following two washes with cold staining buffer, cells were resuspended in 90 μL of buffer and incubated with 10 μL of Streptavidin MicroBeads (Miltenyi Biotech, MD, USA, catalog #130-048-101) for 20 min at 4 °C. Magnetic separation was performed using LS columns with a manual MACS separator (Miltenyi Biotech) according to the manufacturer’s protocol.

The mCD45^−^hCD3^−^hCD19^−^ cell population underwent subsequent positive selection through incubation with biotinylated anti-human CD45 antibody (0.5 μg/10^6^ cells in 100 μL staining buffer) for 20 min on ice. After washing, the cells were incubated with Streptavidin MicroBeads (10 μL/10^6^ cells) and separated using MS columns as per the manufacturer’s instructions.

Following magnetic separation, cell concentrations were quantified using a hemocytometer and adjusted to 1 × 10^6^ cells/mL in 1× PBS containing 0.04% bovine serum albumin. Single-cell suspensions were immediately processed for 10x Genomics Chromium Single Cell 3′ Library construction (10x Genomics, CA, USA catalog #PN-1000268) according to the established protocols.

### 4.6. Single-Cell RNA Sequencing Library Preparation

The single-cell transcriptome library was prepared per the manufacturer’s recommendations. Briefly, single cells were loaded onto a 10X Genomics Chromium chip (10X Genomics) to generate single-cell Gel Bead-in-emulsion (GEM). scRNA-Seq libraries were prepared using GemCode Single-Cell Gel Bead and Library Kit (10X Genomics). GEM reverse transcription was performed with the following conditions: 55 °C for 2 h, 85 °C for 5 min; held at 4 °C. After reverse transcription, GEMs were broken, and the single-strand cDNA was cleaned up with DynaBeads MyOne Silane Beads (Thermo Fisher Scientific, MA, USA) and the SPRIselect Reagent Kit (0.6× SPRI; Beckman Coulter, IN, USA). cDNA was amplified with the following condition: 98 °C for 3 min; 14 cycles of 98 °C for 15 s, 67 °C for 20 s, and 72 °C for 1 min; 72 °C for 1 min; held at 4 °C. The cDNA product was cleaned up using the SPRIselect Reagent Kit (0.6× SPRI; Beckman Coulter, IN, USA). Indexed sequencing libraries were constructed with the reagents in the GemCode Single-Cell 3′ Library Kit (10X Genomics). Sequencing was performed on Illumina NextSeq 500 with NextSeq 500/550 v2.5 kits.

### 4.7. Quality Control and Data Analysis of scRNA-Seq

Raw sequencing reads were processed and mapped to the hg19 human reference transcriptome using the Cell Ranger version 1.1.0 pipeline from 10X Genomics for individual scRNA-Seq datasets. The Seurat V4 R package was used to analyze the scRNA-Seq data.

### 4.8. Cell Clustering Analysis

High-quality transcriptomes that passed the quality control criteria (200–2500 genes and <15% mitochondrial genes) were log-normalized, scaled, and subjected to PCA spaces using the top 2000 highly variable genes (HVGs). Non-linear dimension reduction algorithms, including UMAP or t-SNE, were utilized to visualize the transcriptomes in 2D space. The Louvain algorithm was used to perform unsupervised clustering, and the assigned cell cluster was annotated based on their expression of canonical immune cell subtype markers. All the functions used for clustering analysis were wrapped in the Seurat V4 toolkit [45]. Cell type-specific feature gene acquisition or comparing DEGs of a cell type in different treatment groups was performed using the *FindAllMarkers* or *FindMarkers* function in the Seurat V4 toolkit.

### 4.9. Cell Type Correlation Analysis

The human spleen scRNA-seq data generated by Madissoon et al. [22] were obtained from https://www.tissuestabilitycellatlas.org (accessed on 14 March 2025). scRNA-seq data of innate immune cells from human and humanized mice were first merged and preprocessed as mentioned above. The merged profiles were then subjected to the batch-corrected PCA space using the Harmony algorithm [46], followed by t-SNE analysis. The merged profiles were integrated following the Seurat CCA integration guide (https://satijalab.org/seurat/articles/integration_introduction (accessed on 14 March 2025)), and then phylogenetic analysis was performed and visualized using the BuildClusterTree and PlotClusterTree functions in the Seurat V4 toolkit. For Pearson’s correlation analysis, scRNA-seq profiles of highly variable genes identified were first selected and then converted into pseudo-bulk profiles by calculating the average expression of the HVGs in each innate immune cell subpopulation. A Z-score scaling procedure was then performed on the pseudo-bulk profiles. Pearson’s correlation coefficient was then calculated and visualized using the corrplot function in the R package corrplot.

### 4.10. Gene Clustering Analysis

Feature genes of a particular cell type in different treatment groups were first acquired by performing a one-versus-all Wilcoxson test using the FindAllMarkers function wrapped in the Seurat V4 toolbox. The function was run using default parameters with the expectation that only.pos was set to TRUE and logfc.threshold was set to 0.25. The feature genes were then clustered into 4 modules based on the Mfuzz algorithm. The genes in each module were then enriched for GOBP pathways. Enriched pathways with *p*-value > 0.05 or that appeared in more than 1 module were then filtered out. The clustering, enrichment, and visualization were performed using corresponding functions wrapped in the R package ClusterGVis (https://github.com/junjunlab/ClusterGVis (accessed on 17 March 2025)).

### 4.11. Gene Set Enrichment Analysis (GSEA)

GSEA was performed using the R package clusterProfiler [47], and the fene sets were obtained from the Molecular Signature Database (MSigDB, https://www.gsea-msigdb.org (accessed on 5 March 2025)).

### 4.12. Flow Cytometry

APC/Cy7-conjugated anti-human CD45 (HI30), BV421-conjugated anti-human CD127 (A019D5), PE/Cy7-conjugated anti-human CD117 (104D2), PerCP-Cy5.5-conjugated anti-human CD19 (HIB19), CD4(SK3), CD14(M5E2), CD16(3G8), CD20(2H7), CD34(581), CD123(6H6), CD11c(Bu15), CRTH2(BM16), PE-conjugated anti-human CD94(DX22), NKG2D(1D11), KIR(UP-R1), KLRG1(SA231A2), IL-22 (2G12A41), APC-conjugated anti-human CD56 (QA17A16), IL-17 (QA18A46), and FITC-conjugated anti-human CD9 (HI9a) were purchased from Biolegend (CA, USA). Pacific orange-conjugated anti-mouse CD45 (30-F11), PE/Texas red-conjugated anti-human CD3 (7D6), PE/Texas red-conjugated anti-human CD4 (RPA-T4), and a LIVE/DEAD Fixable Yellow Dead Cell Stain Kit were purchased from Invitrogen (CA, USA).

For surface marker staining, leukocytes were incubated with antibodies on ice for 30 min and then washed and fixed for analysis. For intracellular cytokine detection, freshly isolated cells were stimulated for 4 h with PMA (50 ng/mL) and ionomycin (1 μM) in the presence of BFA (1 μM). Cells were first stained with surface markers and then fixed and permeabilized with Cytofix/Cytoperm buffer (BD Bioscience, NJ, USA), followed by intracellular staining. Cells were analyzed on a CyAn ADP flow cytometer (Dako, CA, USA). Data were analyzed using Summit 4.3 software (Dako, CA, USA).

### 4.13. Statistics

All experimental data analyses were performed using GraphPad Prism 10.1.0 (GraphPad Software, CA, USA). Two-tailed, unpaired Student’s *t*-tests were employed for the statistical significance query in pairwise comparisons. For multi-group comparisons, data were analyzed by Brown–Forsythe and Welch ANOVA tests, followed by Dunnett’s T3 multiple comparisons test. Results with *p* < 0.05 were considered to be statistically significant. Asterisks resemble significance levels (* *p* < 0.05, ** *p* <0.01, *** *p* < 0.001, **** *p* < 0.0001).

### 4.14. Study Approval

All animal experiments were conducted in strict compliance with the NIH guidelines for laboratory animal housing and care, with protocols approved by the University of North Carolina Institutional Animal Care and Use Committee (Protocol ID 17-071). Human fetal liver tissues (gestational age: 16–20 weeks) were obtained from medically indicated or elective termination of pregnancy via Advanced Bioscience Resources, a nonprofit organization coordinating with outpatient clinics. Written informed consent was systematically obtained from all maternal donors in accordance with clinic regulations. The experimental protocol underwent formal review by the University of North Carolina’s Office of Human Research Ethics, which formally determined that this study does not meet the regulatory definition of human subjects research under the applicable federal guidelines [45 CFR 46.102(d/f) and 21 CFR 56.102(c)(e)(l)].

## Data Availability

All data are available in the manuscript or Supplementary Materials. The RNA-seq data will be deposited according to the guidelines by the journal.

## Supplementary Materials

The following supporting information can be downloaded at: https://www.mdpi.com/article/10.3390/v17081099/s1, Figure S1: Gating strategies for human NK cells from the spleens of humanized mice. Flow cytometry of viable human splenocytes (Y7–mCD45-hCD45+) stained for CD3 and CD56; Figure S2: Gating strategies for human ILC3s from the spleens of humanized mice. Flow cytometry of viable human splenocytes (Y7–mCD45-hCD45+) stained for lineage markers (CD3, CD14, CD16, CD19, CD20, CD123, CD11c, CD34, and CRTH2) and for CD127+CD117+ ILC3s; Table S1: Cluster markers; Table S2: DEGs comparing cells in different groups; Table S3: Top 10 GOBP enrichment of DEGs; Table S4: Clustered genes and enriched pathways in NKs; Table S5: Clustered genes and enriched pathways in ILC3s.

## References

[1] Archin N.M., Sung J.M., Garrido C., Soriano-Sarabia N., Margolis D.M. (2014). Eradicating HIV-1 infection: Seeking to clear a persistent pathogen. Nat. Rev. Microbiol..

[2] Ghosn J., Taiwo B., Seedat S., Autran B., Katlama C. (2018). HIV. Lancet.

[3] Simon V., Ho D.D., Abdool Karim Q. (2006). HIV/AIDS epidemiology, pathogenesis, prevention, and treatment. Lancet.

[4] Deeks S.G. (2011). HIV infection, inflammation, immunosenescence, and aging. Annu. Rev. Med..

[5] Vivier E., Artis D., Colonna M., Diefenbach A., Di Santo J.P., Eberl G., Koyasu S., Locksley R.M., McKenzie A.N.J., Mebius R.E. (2018). Innate Lymphoid Cells: 10 Years On. Cell.

[6] Florez-Alvarez L., Hernandez J.C., Zapata W. (2018). NK Cells in HIV-1 Infection: From Basic Science to Vaccine Strategies. Front. Immunol..

[7] Jackson E., Zhang C.X., Kiani Z., Lisovsky I., Tallon B., Del Corpo A., Gilbert L., Bruneau J., Thomas R., Cote P. (2017). HIV exposed seronegative (HESN) compared to HIV infected individuals have higher frequencies of telomeric Killer Immunoglobulin-like Receptor (KIR) B motifs; Contribution of KIR B motif encoded genes to NK cell responsiveness. PLoS ONE.

[8] Marras F., Nicco E., Bozzano F., Di Biagio A., Dentone C., Pontali E., Boni S., Setti M., Orofino G., Mantia E. (2013). Natural killer cells in HIV controller patients express an activated effector phenotype and do not up-regulate NKp44 on IL-2 stimulation. Proc. Natl. Acad. Sci. USA.

[9] May M.E., Pohlmeyer C.W., Kwaa A.K., Mankowski M.C., Bailey J.R., Blankson J.N. (2020). Combined effects of HLA-B* 57/5801 elite suppressor CD8+ T cells and NK cells on HIV-1 replication. Front. Cell. Infect. Microbiol..

[10] Sugawara S., Reeves R.K., Jost S. (2022). Learning to Be Elite: Lessons From HIV-1 Controllers and Animal Models on Trained Innate Immunity and Virus Suppression. Front. Immunol..

[11] Anderko R.R., DePuyt A.E., Bronson R., Bullotta A.C., Aga E., Bosch R.J., Jones R.B., Eron J.J., Mellors J.W., Gandhi R.T. (2024). Persistence of a Skewed Repertoire of NK Cells in People with HIV-1 on Long-Term Antiretroviral Therapy. J. Immunol..

[12] Mavilio D., Benjamin J., Daucher M., Lombardo G., Kottilil S., Planta M.A., Marcenaro E., Bottino C., Moretta L., Moretta A. (2003). Natural killer cells in HIV-1 infection: Dichotomous effects of viremia on inhibitory and activating receptors and their functional correlates. Proc. Natl. Acad. Sci. USA.

[13] Lichtfuss G.F., Cheng W.J., Farsakoglu Y., Paukovics G., Rajasuriar R., Velayudham P., Kramski M., Hearps A.C., Cameron P.U., Lewin S.R. (2012). Virologically suppressed HIV patients show activation of NK cells and persistent innate immune activation. J. Immunol..

[14] Nabatanzi R., Bayigga L., Cose S., Rowland-Jones S., Canderan G., Joloba M., Nakanjako D. (2019). Aberrant natural killer (NK) cell activation and dysfunction among ART-treated HIV-infected adults in an African cohort. Clin. Immunol..

[15] Kloverpris H.N., Kazer S.W., Mjosberg J., Mabuka J.M., Wellmann A., Ndhlovu Z., Yadon M.C., Nhamoyebonde S., Muenchhoff M., Simoni Y. (2016). Innate Lymphoid Cells Are Depleted Irreversibly during Acute HIV-1 Infection in the Absence of Viral Suppression. Immunity.

[16] Kramer B., Goeser F., Lutz P., Glassner A., Boesecke C., Schwarze-Zander C., Kaczmarek D., Nischalke H.D., Branchi V., Manekeller S. (2017). Compartment-specific distribution of human intestinal innate lymphoid cells is altered in HIV patients under effective therapy. PLoS Pathog..

[17] Cheng L., Ma J., Li J., Li D., Li G., Li F., Zhang Q., Yu H., Yasui F., Ye C. (2017). Blocking type I interferon signaling enhances T cell recovery and reduces HIV-1 reservoirs. J. Clin. Investig..

[18] Cheng L., Yu H., Li G., Li F., Ma J., Li J., Chi L., Zhang L., Su L. (2017). Type I interferons suppress viral replication but contribute to T cell depletion and dysfunction during chronic HIV-1 infection. J. Clin. Investig..

[19] Li G., Cheng M., Nunoya J., Cheng L., Guo H., Yu H., Liu Y.J., Su L., Zhang L. (2014). Plasmacytoid dendritic cells suppress HIV-1 replication but contribute to HIV-1 induced immunopathogenesis in humanized mice. PLoS Pathog..

[20] Zhen A., Rezek V., Youn C., Lam B., Chang N., Rick J., Carrillo M., Martin H., Kasparian S., Syed P. (2017). Targeting type I interferon-mediated activation restores immune function in chronic HIV infection. J. Clin. Investig..

[21] Cheng L., Yu H., Wrobel J.A., Li G., Liu P., Hu Z., Xu X.N., Su L. (2020). Identification of pathogenic TRAIL-expressing innate immune cells during HIV-1 infection in humanized mice by scRNA-Seq. J. Clin. Investig..

[22] Madissoon E., Wilbrey-Clark A., Miragaia R.J., Saeb-Parsy K., Mahbubani K.T., Georgakopoulos N., Harding P., Polanski K., Huang N., Nowicki-Osuch K. (2019). scRNA-seq assessment of the human lung, spleen, and esophagus tissue stability after cold preservation. Genome Biol..

[23] Fernandez S., Tanaskovic S., Helbig K., Rajasuriar R., Kramski M., Murray J.M., Beard M., Purcell D., Lewin S.R., Price P. (2011). CD4+ T-cell deficiency in HIV patients responding to antiretroviral therapy is associated with increased expression of interferon-stimulated genes in CD4+ T cells. J. Infect. Dis..

[24] Dunham R.M., Vujkovic-Cvijin I., Yukl S.A., Broadhurst M.J., Loke P., Albright R.G., Wong J.K., Lederman M.M., Somsouk M., Hunt P.W. (2014). Discordance between peripheral and colonic markers of inflammation during suppressive ART. J. Acquir. Immune Defic. Syndr..

[25] Borkar S.A., Yin L., Venturi G.M., Shen J., Chang K.-F., Fischer B.M., Nepal U., Raplee I.D., Sleasman J.W., Goodenow M.M. (2025). Youth Who Control HIV on Antiretroviral Therapy Display Unique Plasma Biomarkers and Cellular Transcriptome Profiles Including DNA Repair and RNA Processing. Cells.

[26] Koopman L.A., Kopcow H.D., Rybalov B., Boyson J.E., Orange J.S., Schatz F., Masch R., Lockwood C.J., Schachter A.D., Park P.J. (2003). Human decidual natural killer cells are a unique NK cell subset with immunomodulatory potential. J. Exp. Med..

[27] Orrantia A., Vazquez-De Luis E., Astarloa-Pando G., Terren I., Amarilla-Irusta A., Polanco-Alonso D., Gonzalez C., Uranga A., Carrascosa T., Mateos-Mazon J.J. (2022). In vivo expansion of a CD9(+) decidual-like NK cell subset following autologous hematopoietic stem cell transplantation. iScience.

[28] Umotoy J.C., Kroon P.Z., Man S., van Dort K.A., Atabey T., Schriek A.I., Dekkers G., Herrera-Carrillo E., Geijtenbeek T.B.H., Heukers R. (2024). Inhibition of HIV-1 replication by nanobodies targeting tetraspanin CD9. iScience.

[29] Martinet L., Smyth M.J. (2015). Balancing natural killer cell activation through paired receptors. Nat. Rev. Immunol..

[30] Houchins J.P., Lanier L.L., Niemi E.C., Phillips J.H., Ryan J.C. (1997). Natural killer cell cytolytic activity is inhibited by NKG2-A and activated by NKG2-C. J. Immunol..

[31] Wada H., Matsumoto N., Maenaka K., Suzuki K., Yamamoto K. (2004). The inhibitory NK cell receptor CD94/NKG2A and the activating receptor CD94/NKG2C bind the top of HLA-E through mostly shared but partly distinct sets of HLA-E residues. Eur. J. Immunol..

[32] Zhang Z., Cheng L., Zhao J., Li G., Zhang L., Chen W., Nie W., Reszka-Blanco N.J., Wang F.S., Su L. (2015). Plasmacytoid dendritic cells promote HIV-1-induced group 3 innate lymphoid cell depletion. J. Clin. Investig..

[33] Zhao J., Cheng L., Wang H., Yu H., Tu B., Fu Q., Li G., Wang Q., Sun Y., Zhang X. (2018). Infection and depletion of CD4+ group-1 innate lymphoid cells by HIV-1 via type-I interferon pathway. PLoS Pathog..

[34] Jarade A., Di Santo J.P., Serafini N. (2021). Group 3 innate lymphoid cells mediate host defense against attaching and effacing pathogens. Curr. Opin. Microbiol..

[35] Serafini N., Di Santo J.P. (2024). Group 3 innate lymphoid cells: A trained Gutkeeper. Immunol. Rev..

[36] Wang Q., Gao H., Clark K.M., Mugisha C.S., Davis K., Tang J.P., Harlan G.H., DeSelm C.J., Presti R.M., Kutluay S.B. (2021). CARD8 is an inflammasome sensor for HIV-1 protease activity. Science.

[37] Hao J., Liu C., Gu Z., Yang X., Lan X., Guo X. (2024). Dysregulation of Wnt/beta-catenin signaling contributes to intestinal inflammation through regulation of group 3 innate lymphoid cells. Nat. Commun..

[38] Bjorkstrom N.K., Strunz B., Ljunggren H.G. (2022). Natural killer cells in antiviral immunity. Nat. Rev. Immunol..

[39] Jabrane-Ferrat N. (2019). Features of Human Decidual NK Cells in Healthy Pregnancy and During Viral Infection. Front. Immunol..

[40] Grulich A.E., van Leeuwen M.T., Falster M.O., Vajdic C.M. (2007). Incidence of cancers in people with HIV/AIDS compared with immunosuppressed transplant recipients: A meta-analysis. Lancet.

[41] Brooks J.T., Kaplan J.E., Holmes K.K., Benson C., Pau A., Masur H. (2009). HIV-associated opportunistic infections--going, going, but not gone: The continued need for prevention and treatment guidelines. Clin. Infect. Dis..

[42] Cao X., Shores E.W., Hu-Li J., Anver M.R., Kelsall B.L., Russell S.M., Drago J., Noguchi M., Grinberg A., Bloom E.T. (1995). Defective lymphoid development in mice lacking expression of the common cytokine receptor gamma chain. Immunity.

[43] Nochi T., Denton P.W., Wahl A., Garcia J.V. (2013). Cryptopatches are essential for the development of human GALT. Cell Rep..

[44] McKenzie A.N.J., Spits H., Eberl G. (2014). Innate lymphoid cells in inflammation and immunity. Immunity.

[45] Hao Y., Hao S., Andersen-Nissen E., Mauck W.M., Zheng S., Butler A., Lee M.J., Wilk A.J., Darby C., Zager M. (2021). Integrated analysis of multimodal single-cell data. Cell.

[46] Korsunsky I., Millard N., Fan J., Slowikowski K., Zhang F., Wei K., Baglaenko Y., Brenner M., Loh P.R., Raychaudhuri S. (2019). Fast, sensitive and accurate integration of single-cell data with Harmony. Nat. Methods.

[47] Wu T., Hu E., Xu S., Chen M., Guo P., Dai Z., Feng T., Zhou L., Tang W., Zhan L. (2021). clusterProfiler 4.0: A universal enrichment tool for interpreting omics data. Innovation.

