# Supplementary material for: Persistent Type I Interferon Signaling Impairs Innate Lymphoid Cells During HIV-1 Infection Under Suppressive ART
Source: Viruses. 2025 Aug 8;17(8):1099. doi: 10.3390/v17081099 (PMC12390710; doi:10.3390/v17081099)
Supplement: Supplementary file 1 [file viruses-17-01099-s001.zip › viruses-3744813-supplementary/viruses-3744813-supplementary/viruses-3744813-supplementary-figure.pdf]

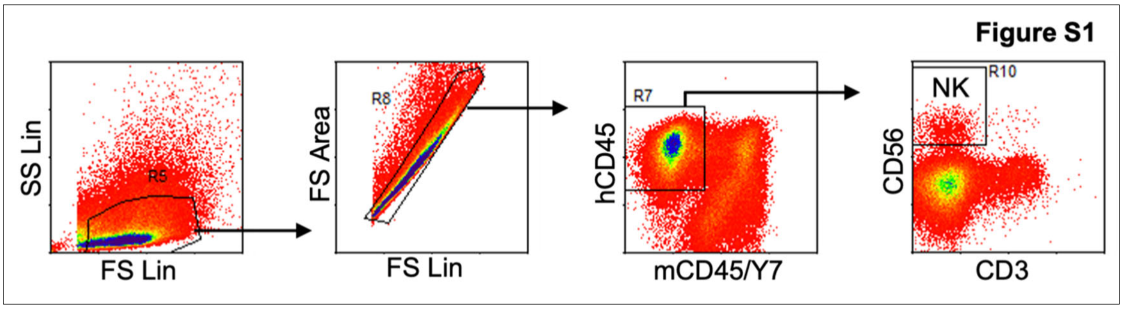

Figure S1. Gating strategies for human NK cells from the spleens of humanized mice. Flow cytometry of viable human splenocytes (Y7⁻mCD45⁻hCD45⁺) stained for CD3 and CD56.

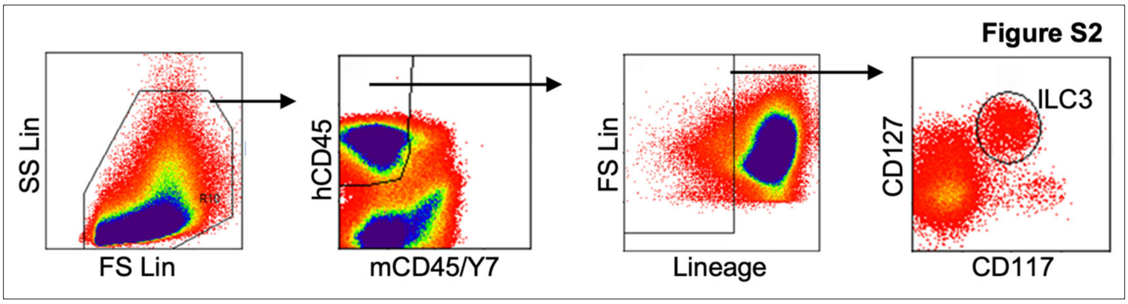

Figure S2. Gating strategies for human ILC3s from the spleens of humanized mice. Flow cytometry of viable human splenocytes (Y7⁻mCD45⁻hCD45⁺) stained for lineage markers (CD3, CD14, CD16, CD19, CD20, CD123, CD11c, CD34, and CRTH2) and for CD127⁺CD117⁺ ILC3s.
